# *In vivo* and *in vitro *effects of *Salsola collina* on gastrointestinal motility in rats

**DOI:** 10.22038/IJBMS.2019.40613.9605

**Published:** 2020-03

**Authors:** Shasha Wang, Meixing Yan, Yaoyao Guo, Runzhou Sun, Hong Jin, Yanling Gong

**Affiliations:** 1 Department of Pharmacy, College of Chemical Engineering, Qingdao University of Science & Technology, Qingdao, China; 2 Qingdao Women and Children’s Hospital, Qingdao, China

**Keywords:** Atropine, Epinephrine, Gastrointestinal motility, Gastrointestinal hormones, Rat

## Abstract

**Objective(s):**

*Salsola collina *is widely distributed along the Bohai coast and consumed as an edible plant by native residents. We have found surprisingly that *S. collina* extracts promoted gastrointestinal motility in mice previously. In the present study, effects of *S. collina* on gastrointestinal motility in rats and its underlying mechanism were explored.

**Materials and Methods:**

*In vivo*, different fraction extracts from *S. collina *were prepared and the effects on gastric emptying and small intestinal propulsion in normal rats were measured. Plasma ghrelin (GRL), motilin (MTL), gastrin (GAS) and vasoactive intestinal peptide (VIP) and expressions of GRL receptor (GHSR), MTL receptor (MTLR), VIP receptor 2 (VIPR2) in the duodenum were also detected. *In vitro*, gastric antrum strips were prepared and activities of different extracts on gastric smooth muscle contractions were evaluated.

**Results:**

Results showed that the ethyl acetate extract (EAE) was the most effective fraction to promote gastric emptying and intestinal propulsion, showing a dose-dependent manner. EAE increased plasma GRL and GAS, elevated GHSR expression and restrained VIPR2 expression in the duodenum. *In vitro*, EAE promoted contraction of normal gastric antrum strips as well as relaxed strips induced by atropine.

**Conclusion:**

These data indicate that EAE has a significant prokinetic activity via a mechanism that mainly involves in modulating plasma GRL and GAS, expressions of GSHR and VIPR2 in the duodenum and activating M-cholinergic receptor. Our study provides a pharmacological basis for the use of *S. collina* extract in treating gastrointestinal motility disorders.

## Introduction

Gastrointestinal diseases, the major causes of human ill-health, are common public health problems throughout the world (1). Indigestion and constipation, in particular, are commonly prevailing disorders. Indigestion is known to affect approximately 11-29.2% of the population, while the prevalence of constipation is up to 27% (2, 3). Dietary and lifestyle measures are first-line treatment for gut motility disorders world widely (4). The second choice of therapy is the prokinetics and laxatives which would become less efficient with long-term use (5). Recently, medicinal plants have attracted attention to treat gastrointestinal disorders such as indigestion and constipation, since there is an increasing evidence that multiple constituents found in medicinal plants have the potential synergies (6, 7). Medicinal plants are considered relatively safe and effective in prolonged use, especially in patients with chronic gut motility disorders.

*Salsola collina*, a perennial herb of the Chenopodiaceae, is widely distributed in China, and the fresh *S. collina* is consumed as an herbal drink or medicine to treat diseases including hypertension, headache and vertigo (8, 9). It has been reported that the ethanol extract of *S. collina* has antioxidative, anti-cancer and antihypertensive activities (10, 11). In addition, an aqueous extract from *S. collina* is an effective means for the prophylaxis of cholelithiasis (12). Although the use of *S. collina* has a long history in traditional Chinese medicine, there are few studies on its pharmacological effect involving in gastrointestinal motility as well as the possible underlying mechanism.

The aim of the present study was to investigate the activities of different extracts from *S. collina* on gastrointestinal motility both *in vivo *and *in vitro*. At the same time, the underlying mechanisms were explored here.

## Materials and Methods

***Reagents and chemicals***

Standards for ferulic acid and vanillic acid (98.0%) were purchased from Chengdu Derick Biotenhnology Co., Ltd. (Chengdu, China). Atropine and epinephrine were purchased from Sigma Chemical Co., Ltd. (St. Louis, MO, USA). The Krebs solution contained (mM, pH 7.4): NaCl, 118; KCl, 4.8; KH_2_PO_4_, 1.2; MgSO_4_, 1.2; CaCl_2_, 2.5; NaHCO_3_, 25; glucose, 11 (13). All other reagents were obtained from Sinopharm Chemical Reagent Co., Ltd. (Shanghai, China).

***Plant material and preparation of S. collina***

*S. collina* was purchased from Qizhou medicine Hall (Hebei, China) and identified as the over-ground part of *S. collina* by Professor Hong Jin (Qingdao University of Science and Technology, China). *S. collina* was powdered and extracted with 70% ethanol by heating reflux method for 2 hr and 3 times. The extract was evaporated under reduced pressure to remove ethanol at a temperature below 60 ^°^C, then suspended in water and partitioned successively with petroleum ether, ethyl acetate and n-butanol. The extracts obtained from each step was evaporated to dryness separately and redissolved in saline before use. Four extracts from *S. collina* were petroleum ether extract (PEE), ethyl acetate extract (EAE), n-butanol extract (NBE) and remainder after extraction (RAE), respectively.

***Animals***

Male Sprague Dawley rats (250-270 g) were provided by Qingdao Daren Fortune Animal Technology Co., Ltd. (approval number: SCXK (Jing) 2016-0002) housed in cages in a temperature-controlled room (22±2 ^°^C) and on a 12-hr light-dark cycle, with free access to standard animal chow and tap water. All the protocols were approved by the Animal Care and Use Committee at Qingdao University of Science and Technology (approval number: ACQUST-2017-025).

***HPLC analysis of EAE***

HPLC was performed on an Agilent 1220 Infinity LC System (Agilent Technologies, USA), and agilent Eclipse XDB C18 (4.6×250 mm, 5 μm; Agilent, USA) at 30 ^°^C. Elution solvents were 0.01% (v/v) phosphoric acid in water (A) and acetonitrile (B), with a gradient elution program was set as follows: 5% (B) for 0-9 min, 5-10% (B) for 9-15 min, 10-15% (B) for 15-20 min, 15-20 (B) for 20-30 min, 20-30 (B) for 30-40 min, 30-20 (B) for 40-50 min, 20-15 (B) for 50-60 min. The flow rate was set at 1.0 ml/min. Chromatograms were acquired with a UV detector at 290 nm.

***In vivo experimental design***

*Different extracts of S. collina in rats*

The effect of different extracts on gastrointestinal motility in rats was observed. Rats were randomly divided into six groups (n=8): control group, PEE group, EAE group, NBE group, RAE group, domperidone group. The rats in PEE, EAE, NBE and RAE groups, PEE, EAE, NBE, RAE (40 mg/kg) were given by intragastric (IG) administration once a day for 7 days. In the positive group, the rats were given 3 mg/kg of domperidone for 7 days. In the control group, the rats were given 0.9% saline solution for 7 days. The gastric emptying rate and intestinal transit rate of each rat was measured at 30 min after the last administration.

*Different doses of EAE in rats*

Rats were divided into five groups (n=8) randomly. The control and Dom groups were treated as mentioned above. The rats in low-dose-EAE group (L-EAE), middle-dose-EAE group (M-EAE), and high-dose-EAE group (H-EAE) were administered EAE for 7 days at doses of 20, 40, 80 mg/kg (IG), respectively. The gastric emptying rate and intestinal transit rate was measured at 30 min after the last treating.

*Test of gastric emptying rate and intestinal transit rate*

Rats were fasted for 24 hr before the experiments. 1.5 ml of 0.05% (w/v) phenol red suspended in 1.5% aqueous methylcellulose solution was administered to rats (IG). Twenty min later, the blood was obtained from abdominal aorta. Immediately, the rats were sacrificed and the whole stomach and intestine tissues were taken out carefully. The stomachs were cut into several pieces and homogenized in 100 ml of 0.1M NaOH. The suspension was settled for 1 hr at room temperature. And then 5 ml of the supernatant was precipitated with 0.5 ml of 20% trichloroacetic acid and centrifuged at 3000 rpm for 10 min. The supernatant was mixed with 4 ml of 0.5M NaOH, and the absorbance was measured at 560 nm. Gastric emptying (%)=(1-amount of phenol red recovered from test stomach/ average amount of phenol red recovered from standard stomachs)×100 (14). The small intestine was stretched on a white paper. The whole length and the pushing distance of phenol red solution were measured accurately. The rate of intestinal transit was expressed as the ratio between the distance travelled by phenol red solution and the total length of intestine (15). Meanwhile, the duodenal was washed with saline on the ice and put into liquid nitrogen immediately for Western blot assays.

*Mearsurement of gastrointestinal hormone in plasma*

Blood samples were collected in tubes containing EDTA, and centrifuged at 2500 rpm for 15 min at 4 ^°^C. All prepared plasma was stored at -80 ^°^C to determine GRL, MTL, GAS and VIP using an ELISA kit (Nanjing Jiancheng Bioengineering Institute, Nanjing, China.)

*Detection on expressions of the relevant gastrointestinal hormone receptors*

 After homogenized at 4 ^°^C with 1 mM EDTA and 2.5 ml cell lysate, the proteins (70 μg) were separated from the duodenum by SDA-PAGE and transferred to positively charged nylon membranes (Pall corporation, NY, USA). The blots were probed with rabbit antibodies of anti-GHSR, anti-MTLR, anti-VIPR2 (dilution 1:200) overnight at 4 ^°^C. The secondary antibodies were goat anti-rabbit IgG-HRP (1:5000; Nanjing Jiancheng Biotechnology institute, Nanjing, China) and the blots were washed with Tris-Buffered Saline and Tween 20 (TBST). The bands were observed using an enhanced chemiluminescence substrate (Amersham Bioscience, Buckinghamshire, UK) and quantified with an image analysis program (Image Gauge v3.12 software, Fujifilm, Tokyo, Japan). Protein concentration was determined using the BCA Protein Assay Kit (16).

***In vitro*** ***experimental design***

*Preparation of gastric antrum strips*

The rats were deprived from food but not water 24 hr prior to experiments, and then stunned by head-striking. The whole stomach was quickly removed and placed in Krebs solution. After removing the mucosa by blunt dissection, gastric antrum strips with approximately 2 cm length were prepared by cutting parallel to the long axis of the tissue and each end was attached by a thread. Then the strips were suspended in tissue chambers filled with Krebs solution at 37±0.5 ^°^C, bubbled with a mixture of oxygen (95%) and carbon dioxide (5%) (17). The strips were mounted under an initial load of 1.0 g, and equilibrate for 1 hr with washout every 20 min before starting the experiment (18).

*Test of the spontaneous contraction on normal gastric antrum muscle strips*

After equilibration for 1 hr, PEE, EAE, NBE and RAE (0.1, 0.2, 0.4 mg/ml) were added to the Krebs solution individually and muscle contractions were recorded. The amplitude of contractions occurring after administration of each concentration of PEE, EAE, NBE, and RAE were determined for 10 min. Relative changes in contractile responses induced by the different concentrations of PEE, EAE, NBE, and RAE relative to the basal levels (before treatment with those extract) were calculated as percentages.

*Test of the spontaneous contraction in relaxed gastric antrum muscle strips*

To investigate the underlying mechanisms of the EAE induced effects on spontaneous contractions, atropine (10^-6^ mol/l) or epinephrine (10^-6^ mol/l) solution was appended into the bath and the contraction curves were recorded. Then the EAE was also added to the bath to 0.4 mg/ml and the amplitude changes of spontaneous contraction were recorded.

***Statistical analysis***

Data analysis was performed using SPSS 23.0 system. The data of each group were expressed in means±SD and analyzed using one-way analysis of variance (ANOVA) followed by Dunnett’s test. *P*-value< 0.05 was considered statistically significant.

## Results

***HPLC analysis of EAE***

**Figure 1.**
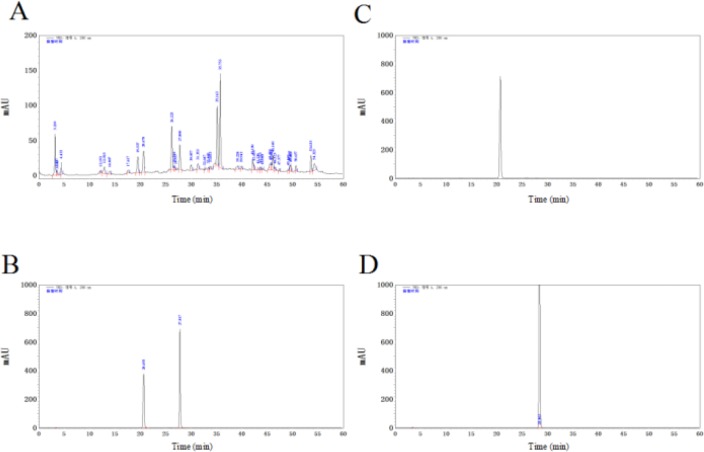
HPLC chromatogram of EAE. HPLC chromatogram of the EAE extract (A); HPLC chromatogram of the mixture of reference samples (B); HPLC chromatogram of vanillic acid (C); HPLC chromatogram of ferulic acid (D). EAE: ethyl acetate extract, HPLC: high-performance liquid chromatography

HPLC analysis of EAE was illustrated in Figure 1. The peak of vanillic acid and ferulic acid in the extract (Figure 1A) were identified according to the retention times of their reference samples (Figure 1C-D) in HPLC.

***Effects of different extracts on gastric emptying and small intestinal motility in rats***

**Figure 2:**
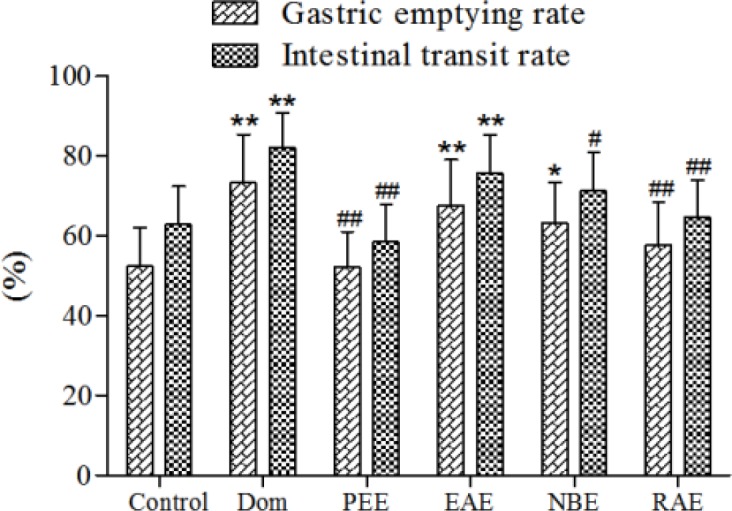
Effects of different extracts on gastric emptying and small intestinal motility in rats. PEE, EAE, NBE, RAE (40 mg/kg) were given by i.g. administration once a day for 7 days, Dom (3 mg/kg) was positive group and the rats were given 0.9% saline solution in the control group. Data are represented as the mean±SD. **Compared with control, *P*-value<0.01; *compared with control, *P*-value< 0.05.##Compared with Dom, *P*-value<0.01; #compared with Dom, *P*-value<0.05. Dom: domperidone group; PEE:petroleum ether extract; EAE: ethyl acetate extract; NBE: n-butanol extract ; RAE: remainder after extraction

To investigate the effects of PEE, EAE, NBE, RAE, we examined the effects of gastric emptying and small intestinal motility in rats at the same dose of 40 mg/kg. EAE, NBE increased the gastric emptying with the ratio 67.63±11.53%, 63.27±10.17%, respectively (versus 52.39±9.76% in control), while PEE and RAE had no effect (Figure 2). Apparently, EAE had the most conspicuous effect compared to the control group (*P*-value<0.01, Figure 2) and similar to domperidone (73.30±12.04%) (*P*-value>0.05, Figure 2).

EAE significantly enhanced the intestinal propulsion with the ratio of 75.77±9.57% (*P*-value<0.05, Figure 2), and showing no difference with Dom group. In addition, NBE, RAE and PEE had no significant difference with the saline control group (versus 62.90±9.48% in control group, *P*-value > 0.05, Figure 2).

***Effects of EAE on gastric emptying and small intestinal motility in rats***

**Figure 3:**
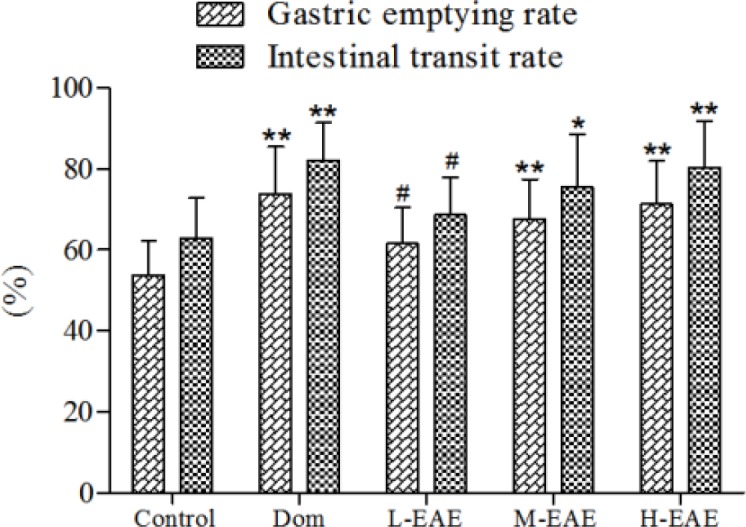
Effects of different doses of EAE on gastric emptying and small intestinal motility in rats. Data are represented as the mean±SD. **Compared with control, *P*-value<0.01; *compared with control, *P*-value<0.05. ##Compared with Dom, *P*-value<0.01; #compared with Dom, *P*-value<0.05

Compared to the control group, M-EAE and H-EAE significantly increased the gastric emptying rate (*P*-value<0.01, Figure 3) and intestinal propulsion (*P*-value<0.05 and *P*-value<0.01 respectively, Figure 3). However, neither of them are as effective as domperidone (*P*-value > 0.05, Figure 3).

***Effects of EAE on gastrointestinal hormone in plasma***

**Figure 4:**
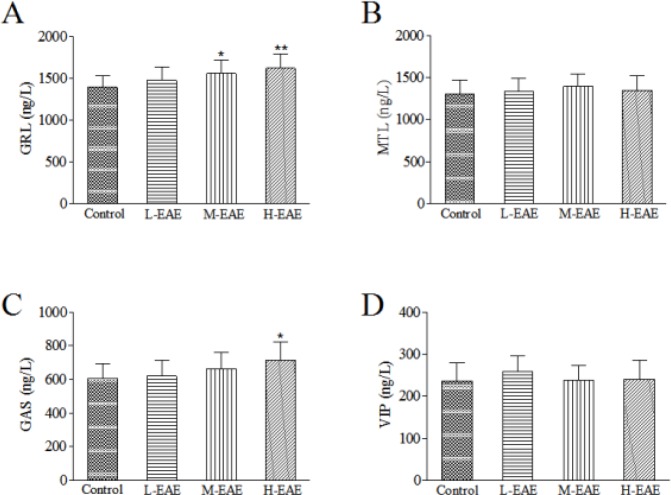
Effect of EAE on gastrointestinal hormone level in plasma. The concentrations of GRL (A), MTL (B), GAS (C) and VIP (D) were measured by ELISA. Data are represented as the mean±SD. **Compared with control, *P*-value<0.01; *compared with control, *P*-value<0.05. GRL: ghrelin, MTL: motilin, GAS: gastrin, VIP: vasoactive intestinal peptide, ELISA: enzymelinked immunosorbent assay

Compared to the control group, plasma GRL and GAS increased in the H-EAE group (*P*-value<0.01 and *P*-value<0.05, respectively, Figure 4), while MTL and VIP had no significant change (*P*-value>0.05, Figure 4).

***Effect of EAE on expressions of the relevant gastrointestinal hormone receptors***

**Figure 5.**
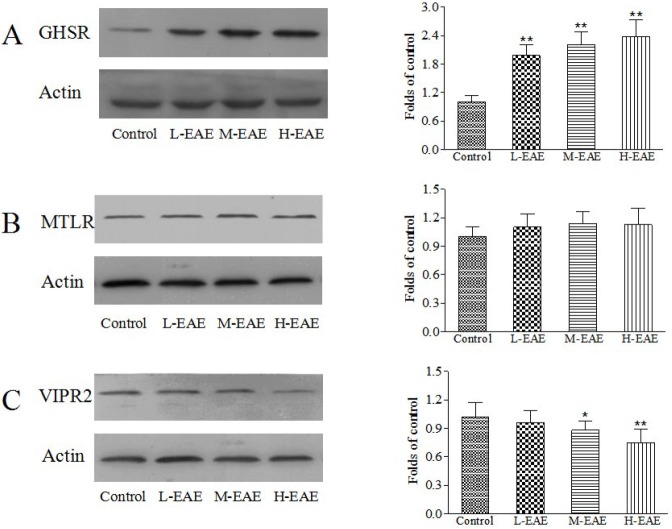
Effect of EAE on the expressions of the relevant gastrointestinal hormone receptors. The expressions for GHSR (A), MTLR (B) and VIPR2 (C) in the duodenum were determined by western blot. Data are represented as the mean±SD. **Compared with control, *P*-value<0.01; *compared with control, *P*-value<0.05

Compared to control group, the expressions of GHSR in the duodenum in different doses of EAE group increased while MTLR had no change (*P*-value>0.05, Figure 5). Besides, the expression of VIPR2 decreased both in M-EAE and H-EAE group (*P*-value<0.05, Figure 5).

***Effect of different extracts on the contractile responses in isolated rat gastric antrum muscle strips***

**Figure 6:**
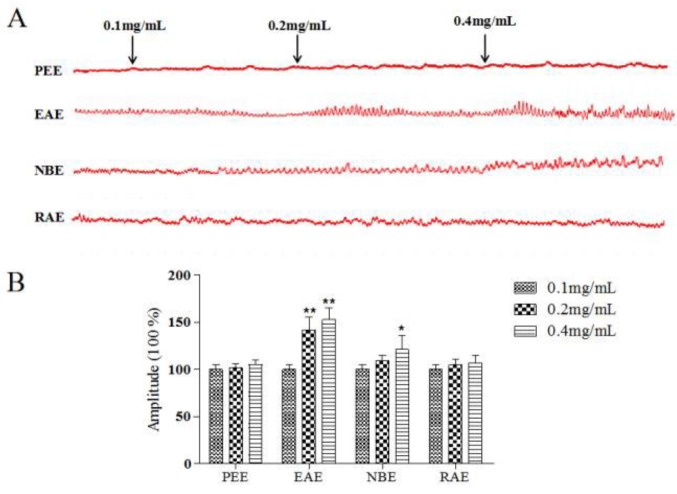
Effects of different extracts on the spontaneous contractility of rat gastric antrum strips. The spontaneous contractility of gastric antrum strips treated with different extracts (A). Amplitude changes in every group (B). The mean contractile amplitude of rat gastric atrum strips in normal contractile state is set as 100% (control). Data are represented as the mean±SD. **Compared with control, *P*-value<0.01; *compared with control, *P*-value<0.05

The effects of PEE, EAE, NBE and RAE on isolated rat gastric antrum muscle motility at doses of 0.1, 0.2, 0.4 mg/ml was observed. EAE showed the strongest effects (*P*-value<0.01, Figure 6) and was tested in the subsequent experiments. NBE (0.4 mg/ml) also had effect (*P*-value<0.05), while PEE and RAE had no effect on isolated rat gastric antrum muscle motility (*P*-value>0.05). EAE increased the strips contraction with a dose-dependent manner in range of 0.1-0.4 mg/ml.

***Effect of EAE on the relaxed gastric antrum muscle strips treated by atropine and epinephrine***

**Figure 7:**
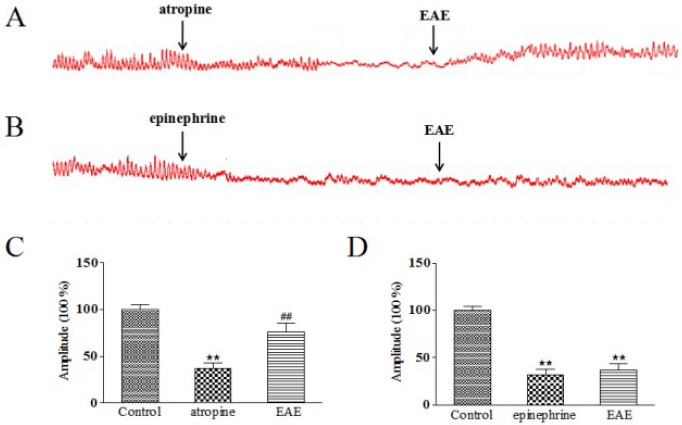
Effects of EAE on atropine and epinephrine induced relaxation of gastric antrum strips. The spontaneous contractility treated with EAE (0.4 mg/ml) after 10 min pretreatment with atropine (10-6 mol/l) and epinephrine (10-6 mol/l), respectively (A, B). Amplitude changes in atropine (C) and epinephrine (D) group. The mean contractile amplitude of rat gastric antrum strips in normal contractile state is set as 100% (control). Data are represented as the mean±SD. **Compared with control, *P*-value<0.01; *compared with control, *P*-value<0.05

The atropine significantly induced relaxation of gastric strips, indicating that atropine inhibited M-cholinergic receptor in gastric antrum. Atropine’s blocking effect was reversed by 0.4 mg/ml of EAE with marked increase of contracting amplitude (*P*-value<0.01, Figure 7), which demonstrated that EAE’s improving function on gastric antrum muscle strips contract was likely involved in activating M-cholinergic receptor. However, the relaxed strips treated by epinephrine had no remarkably change when given with 0.4 mg/ml of EAE (*P*-value>0.05), suggesting that EAE’s exciting effect might had no relation with the adrenergic receptor (Figure 7).

## Discussion

*S. collina* is a pioneer halophyte distributed along the Bohai coast where the soil salt is up to 3%. It is consumed as an edible plant by native residents as it is rich in proteins, microelements, vitamins and antioxidant components (19). Furthermore, its virtues in phyto-remediation for heavy metals and oil in saline soil has been demonstrated in recent years (20, 21). In our previous study, we have found surprisingly that *S. collina* extracts promoted gastrointestinal motility in mice and this discovery drew our great interest to further this research. In the present experiment, the effects of *S. collina* on gastrointestinal motility in rats and its possible underlying mechanism were explored. The results indicate that EAE has a significant prokinetic activity via a mechanism that mainly involves in modulating plasma GRL and GAS, the expressions of GSHR and VIPR2 in the duodenum and activating M-cholinergic receptor. 

Delayed gastric emptying and reduced gastrointestinal motility might resulted in functional gastrointestinal disorders such as indigestion and constipation (22). Natural plants and Chinese herbals are one of the routine treatments for gastrointestinal disorders in China and are gaining much more popularity in other countries (23, 24). Fortunately, we have obtained *S. collina* from plenty of plant resources through extensive screening work. *In vivo*, *S. collina* was confirmed to promote gastric emptying and intestinal propulsion and the ethyl acetate extract was the most effective fraction. Gastrointestinal motility is internally regulated by gastrointestinal smooth muscle electrical activity, nervous system and gastrointestinal hormones. In particularly, gastrointestinal hormones, including GAS, MTL, GRL and VIP, play an important role (25). GAS, synthesized and secreted from G cells locally distributed in the gastric antrum, accelerated gastric emptying and gastrointestinal motility (26). EAE from *S. collina* significantly elevated plasma GAS level in rats. MTL and GRL, synthesized in the upper gastrointestinal tract, are similar in structure and promote gastrointestinal muscle contraction (27). EAE increased plasma GRL and GRL expression in the duodenum while had no significant effect on MTL. Contrary to the above peptides, VIP results in smooth muscles relaxation and gastrointestinal motility inhibition via its specific receptors (28). There are two types of VIP receptor including VIP receptor-1 (VIPR1) and VIP receptor-2 (VIPR2). However, only VIPR2 is abundantly expressed in the gastrointestinal tract (29). In the present study, we revealed that EAE restrained the expression of VIPR2 in the duodenum although plasma VIP had no significant change. Therefore, EAE might increase plasma GRL and GAS, elevate the expression of GHSR and restrain the expression of VIPR2 in the duodenum, which resulted in the acceleration of gastric emptying and intestinal propulsion* in vivo*.

In order to explore the effects and underlying mechanisms of *S. collina* *in vitro*, gastric antrum strips were prepared to test the effects of different fractions from *S. collina*. It revealed that different fractions from *S. collina* stimulated the contraction of gastric antrum strips and the intensity was as follows: EAE>NBE>RAE>PEE. The stimulation of EAE was the strongest, which was consistent with the *in vivo* results. Furthermore, EAE reversed the blocking effect of atropine rather than epinephrine. Atropine, a non-selective muscarinic antagonist, causes an obvious relaxation of gastric antrum strips via blocking the M-muscarinic receptor. Simultaneously, epinephrine activates β_2_-adrenegic receptor in the gastric smooth muscles and results in a relaxation effect. Integratively, the stomach receives a dense parasympathetic vagal innervations arised from the dorsal motor nucleus of the vagus (DMV) in the brainstem (30). However, no reverse effect of EAE on that of epinephrine was observed. Taken together, the stimulation of EAE on gastric antrum strips might likely involve in activating M-cholinergic receptor.

EAE: ethyl acetate extract

EAE: ethyl acetate extract

EAE: ethyl acetate extract

EAE: ethyl acetate extract

EAE: ethyl acetate extract

## Conclusion

Our results suggest that EAE from *S. collina* has a significant prokinetic activity via a mechanism that mainly involves in modulating plasma GRL and GAS, the expressions of GSHR and VIPR2 in the duodenum and activating M-cholinergic receptor. These findings provide a pharmacological basis for the use of an extract of *S. collina* in the intervention of gastrointestinal motility disorders. The potential application value remains for further exploration.
